# The use of tranexamic acid to reduce blood loss in uncemented total hip arthroplasty for avascular necrosis of femoral head: a prospective blinded randomized controlled study

**DOI:** 10.1186/s42836-019-0012-6

**Published:** 2019-11-21

**Authors:** Javahir A. Pachore, Vikram Indrajit Shah, Sachin Upadhyay, Kalpesh Shah, Ashish Sheth, Amish Kshatriya

**Affiliations:** 1 Department of Hip Arthroplasty, Shalby Hospitals, Ahmedabad, Gujarat India; 2 Department of Knee and Hip Arthroplasty, Shalby Hospitals, Ahmedabad, Gujarat India; 3 Department of Orthopaedics, NSCB Medical College, Jabalpur, MP India; 4 Department of Trauma, Joint Replacement and Minimal Invasive Surgery, Shalby Hospitals Jabalpur, Jabalpur, Madhya Pradesh India

**Keywords:** Tranexamic acid, Osteonecrosis, Total hip arthroplasty, Deep vein thrombosis

## Abstract

**Background:**

The purpose of this prospective, double-blinded, randomized controlled study is to assess the efficacy of administration of intravenous tranexamic acid (TXA) for reducing blood loss in uncemented total hip arthroplasty (THA) for the treatment of osteonecrosis of femoral head.

**Methods:**

Between April 2012 and March 2014, 73 patients with avascular necrosis of femoral head were treated in our center. The patients were randomized and allocated to study group (*n* = 36; treated with TXA) and control group (*n* = 37). Intra- and postoperative blood loss, blood transfusion, and incidence of deep vein thrombosis were assessed. A *p* value less than 0.05 was considered statistically significant.

**Results:**

The intraoperative, postoperative, and total (clinical method and Gross’ formula) blood loss were significantly greater in the control group (*p* < 0.05). On the first, second, and third postoperative days, the levels of hemoglobin and hematocrit were significantly better in the study group (*p* < 0.05). There was a significantly greater number of patients who required blood transfusion in the control group (*p* = .027). Deep vein thrombosis was not found in either group.

**Conclusions:**

A single dose of TXA used preoperatively may minimize intraoperative, postoperative, and total blood loss in uncemented THA for the treatment of osteonecrosis of femoral head, and may not increase the risk of prothrombotic complications.

## Background

Total hip arthroplasty (THA) may cause postoperative blood loss that may necessitate blood transfusion [1, 2]. However, allogenic reactions due to blood transfusion are possible, which may put the patient at the risk of severe complications, and add more cost to the treatments [3–5]. Several strategies have been devised to reduce perioperative blood loss during THA [6]. The use of tranexamic acid (TXA) can reportedly reduce blood loss and thereby decrease blood transfusion after THA [2, 7].

TXA is a synthetic derivative of lysine that exerts its antifibrinolytic effect by blocking the lysine binding sites of plasminogen, which ultimately blocks the degradation of fibrin [8]. This process may also potentially enhance the risk of venous thromboembolic events (VTEs) by promoting thrombosis [9]. Most randomized controlled trials evaluating the efficacy of tranexamic acid during THA included cohorts with a diagnosis of osteoarthritis or those with either osteoarthritis or osteonecrosis of femoral head [4, 10–17]. In contrast to Caucasian population, in India total hip arthroplasty is more commonly performed for avascular necrosis of hip than for primary osteoarthritis [18]. Compromised subchondral microcirculation and ischemia through distinct underlying pathophysical mechanism leading to osteonecrosis [19]. We found no randomized controlled trial evaluating the efficacy of intravenous tranexamic acid in reducing perioperative blood loss and need for blood transfusions after uncemented THA for a specific diagnosis of osteonecrosis of the femoral head only and this was the aim of our study. We also wanted to find out if there is any high incidence of DVT in this group. We hypothesized that a single pre-incisional dose of intravenous tranexamic acid reduces blood loss associated with uncemented THA for osteonecrosis of femoral head.

## Materials and methods

The institutional review boards of the participating hospitals reviewed the study and approved the protocol. Informed consent was obtained from each patient.

Between April 2012 and March 2014, patients undergoing uncemented THA for osteonecrosis of femoral head were considered for the study. Participants were recruited into the study, and randomized into two groups: treatment and control group. Study was designed as a 1:1 case control study. Our eligibility criteria were unilateral involvement; avascular necrosis of femoral head with moderate to severe arthritis; hip pain interfering with daily living; and primary THA. Patients were excluded if they had one of the following: 1) mild arthritis, rheumatoid arthritis, post-traumatic arthritis; 2) thrombocytopenia (platelet count < 100,000/mm3; 3) anaemia (hemoglobin (Hb) < 11 g%; packed cell volume < 33%); 4) under thrombolytic or anticoagulant therapy; 5) haemorrhage or haemorrhagic diathesis; 6) hypersensitivity to tranexamic acid; 7) a history of administration of NSAIDs within 1 week; 8) a history of deep vein DVT/pulmonary embolism (PE); 9) renal dysfunction; 10) major illness and health condition such as severe cardiac disorders; 11) previous ipsilateral hip surgery such as synovectomy, decompression, grafting, osteotomy, and fracture fixation; 12) body mass index (BMI) > 30; 13) decline to participate; 14) unwilling to receive any surgical intervention; 15) bilateral involvement; and 16) osteonecrosis secondary to sickle cell anaemia or renal transplant.

**Fig. 1:**
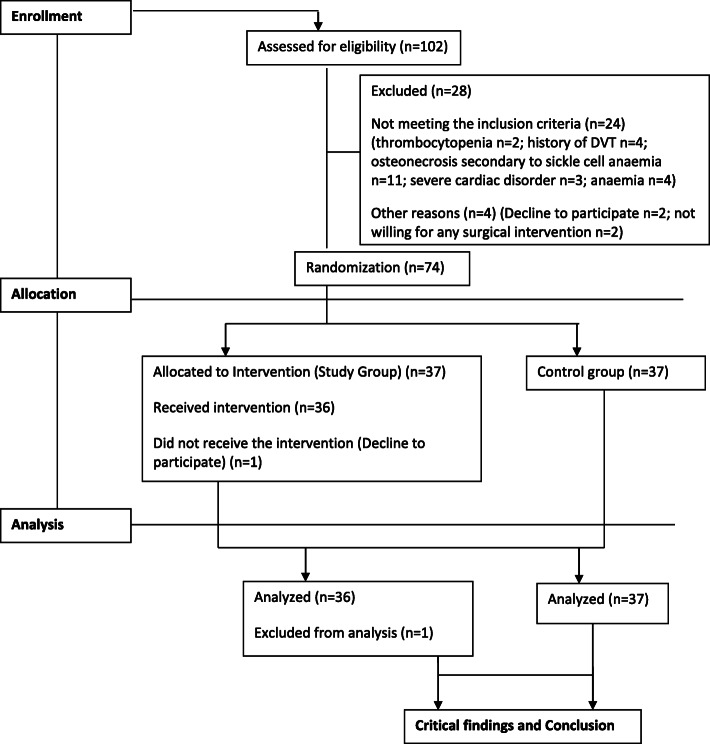
Consort flow chart

Sample size was estimated using formula of simple random sampling for infinite population. Assumptions were considered based on the difference in mean volume of blood loss in treatment [16]. The power analysis determined that the study comprising a minimum of 72 patients required 95% confidence intervals (5% ɑ), 80% power, and 0.1 absolute precision (marginal error). Out of 102 patients, 74 were selected, randomized, and allocated to a study group (*n* = 37) and a control group (*n* = 37). The consort flow chart for the study is shown in Fig. 1.

### TXA administration

Patients in study group and control group received TXA or nothing, respectively, which were packaged in envelopes marked with numbers. The envelopes were randomly drawn by a third person who did not participate in the treatments and were blinded to the sample envelope numbers. The envelopes were handled to the anaesthetists. The surgeons and assessors were blinded. The sample code was not broken until the study was completed. Patients in the study group received a single dose of intravenous TXA (10 mg/ kg body weight, maximum dose: 1 g) 15 min prior to skin incision. Patients in the control group did not receive any drug. All patients were operated on by a single experienced surgeon in lateral position using posterior approach thus ensuring a consistent surgical technique. Intraoperative haemostasis was completely achieved using electrocoagulation. All patients received spinal anaesthesia for surgery by the same anaesthetist. A closed suction drainage system was used in all cases and was removed in 48 h.

### Blood loss calculation

Intraoperative blood loss was measured using an electric weighing scale with an accuracy of 0.1 mg (Alexandra Scale Pvt. Ltd., Gujarat, India). All blood-stained mops and the blood in the suction cylinders were included in the blood loss. The postoperative blood loss was calculated using two methods. First, the blood loss was determined based on the blood collected from the suction drain 12, 24, and 48 h after surgery. Second, the blood loss was estimated based on hematocrit balance using Gross’ formula [20], which included the blood loss of extravasation in the tissues. Gross’ formula are as follows:

$$ Estimated\kern0.17em blood\kern0.17em loss= Estimated\kern0.17em blood\kern0.17em volume\times \left( initial\kern0.17em hematocrit- final\kern0.17em hematocrit\right)/ mean\kern0.17em hematocrit $$

$$ Estimated\kern0.17em blood\kern0.17em volume= body\kern0.17em weight(kg)\times 70 ml/ kg $$

The total blood loss (clinical) was determined by adding intraoperative and postoperative blood loss.

### Laboratory data collection

The levels of hemoglobin and hematocrit concentration, as well as bleeding time, clotting time, prothrombin time, and activated partial thromboplastin time, were tested at admission as the baseline. The parameters on the first and second postoperative days were also obtained. A uniform blood transfusion protocol was used in both groups. Postoperative blood transfusion was given if the level of haematocrit was less than 27% of normal range. Intraoperative, postoperative, and total blood loss (both clinical method and Gross’ formula method were used) were the primary analytic focus.

### Patient managements

Doppler ultrasound was performed prior to surgery and on the 5th postoperative day to rule out DVT. All patients were given anti- thromboembolic stockings for 6 weeks. Chemoprophylaxis for DVT and PE was not used. All patients were ambulated partial weight bearing with support postoperatively as soon as they were comfortable for the first 3 weeks, which was followed by full weight bearing thereafter.

### Statistical analysis

Demographic data were presented as mean ± standard deviation (SD) and range. During the analysis, numerically-coded categorical variables were cross tabulated, and Chi square or Fisher’s exact test was applied as required. If the frequency was less than five, a Fisher’s exact *p* value was used; and Pearson’s Chi square tests were used for other analyses. Student *t* test was used to test the differences between two independent means. The Shapiro-Wilk test was used to test normality. When the variable significantly deviated from a normal distribution, a log transformation was performed for statistical comparison. Analyses were performed using a s tatistical software package (SPSS for Windows software, version 22.0, SPSS, Inc., Chicago, IL, USA).

## Results

There were no significant differences between the two groups with regard to demographic or preoperative characteristics (*p* > 0.05) (Table 1). Intraoperative and postoperative blood loss, changes in the levels of hemoglobin and hematocrit, the amount of blood transfusion and complications are shown in Table 2. In the study group, one patient who refused to receive allocated intervention after randomization was excluded from the final analysis. A total of 73 patients were available for final analysis.

**Table 1:** Patient demographics and preoperative characteristics

Characteristics	Study group (Mean ± SD) (*n* = 37)	Control group (Mean ± SD) (*n* = 37)	*p* value
Age (years)	48.5 ± 13.5	52.2 ± 11.4	0.2
Sex (Male: female)	24: 13	23: 14	0.8
BMI	26.2 ± 1.5	26.8 ± 1.6	0.1
Pre-op Hb (g/100 mL)	13.2 ± 1.5	13.3 ± 1.4	0.7
Pre-op Hct (%)	36.9 ± 3.8	36.7 ± 3.3	0.8
Cause of osteonecrosis (No. of patients)			
Steroid intake	11	10	0.7
Alcoholism	6	4	
Idiopathic	20	23	

*SD* standard deviation, *BMI* body mass index, *Hb* haemoglobin, *Hct* hematocrit

**Table 2:** Comparison of intra- and postoperative characteristics between the two groups

Parameters	Study group (*n* = 36)	Control group (*n* = 37)	*p* value
Operative time (minutes)^a^	125.8 ± 10.2	128.8 ± 9.4	0.195
Intraoperative blood loss (mL)	230.9 ± 41.6	344 ± 144	< 0.0001
Drain (mL)- 12 h	115 ± 31.2	168 ± 51.3	< 0.0001
24 h	74.2 ± 20.4	136 ± 8.5	< 0.0001
48 h	61 ± 18.6	98.6 ± 35.6	< 0.0001
Total blood loss (clinical method)	481 ± 43.9	737.6 ± 137.8	< 0.0001
Total blood loss- Gross’ formula	484.2 ± 260.9	847 ± 374.5	< 0.0001
Post-op Hb			
Day1	12.1 ± 1.4	11.3 ± 1.2	=0.0106
Day 2	11.5 ± 1.1	11 ± 1	=0.046
Drop in Hb			
Day1	1.1 ± 0.4	2 ± 0.7	< 0.0001
Day 2	1.8 ± 1.1	2.4 ± 0.5	=.004
Post-op Hct			
Day 1	34.1 ± 3.4	31.5 ± 3.5	=0.0019
Day 2	33.4 ± 2.5	31.1 ± 1.9	< 0.0001
Drop in Hct			
Day 1	2.7 ± 1.8	5.2 ± 2.1	< 0.0001
Day 2	3.4 ± 1.9	5.5 ± 2.6	0.0002
Blood transfusion (no. of patients)^b^	1	7	.027299
Complications	None	None	1.000

*Hb* Haemoglobin, *Hct* Haematocrit, ^a^Time from induction tol wound closure, ^b^each patient was given one unit of whole blood transfusion

The mean between-group discrepancies in intraoperative blood loss, postoperative blood loss, total blood loss (clinical), and total blood loss (Gross’ formula) were 113 mL, 152 mL, 257 mL, and 363 mL, respectively.

We found that there were significant differences between the two groups with regard to the amount of intraoperative blood loss (*p* < 0.0001) and postoperative drainage (*p* < 0.0001), and total (clinical method and Gross’ formula) blood loss (*p* < 0.0001). We also found significant differences with regard to the levels of hemoglobin concentration immediately after surgery (*p* = 0.0106), and on the first postoperative day (*p* = 0.0019), and on the second postoperative (*p* < 0.0001) day. The levels of hematocrit concentration were also significantly different (*p* < 0.05). The decreased amount of hemoglobin and hematocrit concentration on the first (*p* < 0.0001; *p* < 0.0001) and second (*p* < 0.0001; *p* = 0.0002) postoperative days were significantly different. There were significant difference with regard to the number of patients who received blood transfusion (*p* = .027). Neither deep venous clots nor PEs happened, and nor other complications took place in both groups.

## Discussion

We found TXA can effectively decrease perioperative bleeding and the need for blood transfusions after THA. In a meta-analysis, Zhou et al. [21] found the total blood loss was 305 mL, intraoperative blood was 86 mL, and postoperative blood loss was 177 mL in THA. Another meta-analysis conducted by Huang et al. [7] found that TXA decreased the total blood loss of 389 mL. However, our study included patients undergoing THA for osteonecrosis of femoral head only. The blood loss during THA for osteonecrosis of femoral head is likely to be different from THA for osteoarthritis due to the differences in the degree of marrow edema and synovitis [22]. Clave et al. [11] and Yamasaki et al. [15] reported similar intraoperative blood loss but reduced postoperative blood loss in the tranexamic acid treatment group as compared to the control group. This is in contrast to our findings of reduction in both intraoperative and postoperative blood loss in the tranexamic acid group as compared to the control. Furthermore, they included patients with osteoarthritis only in their study while our study included osteonecrosis of femoral head. Johansson et al. [4], who included patients with osteoarthritis only but performed cemented THAs, also showed no significant difference in intraoperative blood loss in the tranexamic acid group. Rajesparan et al. [6] and Husted et al. [17], with predominantly osteoarthritic patients in their studies, also reported no significant difference in the intraoperative blood loss between tranexamic acid and control groups. It is likely that the indication for THA (osteoarthritis vs. osteonecrosis) might be responsible for the reportedly different pattern of blood loss because the factors, like marrow edema and synovitis, that are likely to influence blood loss, are different in the two pathologies. We administered a single dose of tranexamic acid 15 minutes prior to skin incision. However, the reduction in blood loss was seen intraoperatively and postoperatively up-to 48 h. Surgical trauma and venous stasis causes release of tissue plasminogen activator which initiates fibrinolysis that lasts for an hour [6, 12]. However, the pre-incisional administration of tranexamic acid inhibits fibrinolysis by binding itself to plasminogen. The reduction in intraoperative blood loss as seen in our study might be explained by this effect of tranexamic acid on the coagulation pathway. In our own clinical practice, we have observed reduced intraoperative bleeding and better surgical field in patients receiving tranexamic acid prior to incision. The fibrinolytic inhibition that occurs at about 24 h is due to an increased release of plasminogen activator inhibitor which inactivates tissue plasminogen activator. The sustained effect on reduction in blood loss postoperatively may be in part due to fibrinolytic inhibition and in part due to clot stabilization. This reduces postoperative blood loss after THA in patients receiving tranexamic acid and the result has been widely reported [4, 6, 11, 13, 15, 17]. The two groups in our study were similar (Table 1). Factors that are likely to influence blood loss like age, BMI, gender, and pathology were similar between the two groups. The preoperative Hb and Hct were also similar between the two groups. All patients underwent uncemented THA using the same approach by the same surgeon and anaesthetist to ensure consistency. Both the surgeon and the assessor were blinded to the randomization. Thus, all the factors that are likely to cause bias were controlled. An intravenous dose of 10 mg/kg reportedly maintained therapeutic plasma concentration of tranexamic acid for up to 3 hours [23] and hence this dose was chosen in our study. A higher dose was avoided due to theoretical concern of prothrombotic complications. We used a single dose instead of multiple ones since several studies have confirmed the efficacy of a single dose in reducing blood loss during THA [4, 6]. Benoni et al. have shown that tranexamic acid administered at the end of surgery has no effect on reduction of postoperative blood loss and hence we administered it 15 min prior to incision [24].

Our study focused on a homogenous group of patients undergoing uncemented THA unlike previous studies [6, 12, 17] that have included cemented, uncemented, and hybrid THAs which may behave differently as far as postoperative bleeding is concerned. Uncemented THA behaves differently from cemented or hybrid THA as the femoral canal and possibly the acetabular bony beds are closed off by cement, and the pressurization of cement has a ceasing effect on blood loss from intramedullary circulation. Therefore, postoperative bleeding tends to be higher in the uncemented THA than in the cemented THA due to spontaneous bleeding from intramedullary circulation [15]. With the use of tranexamic acid, there is a concern that it may induce a hypercoagulable state [25]. An interesting feature of our study was the lack of use of chemoprophylaxis against DVT/ PE. Anti-thromboembolic stockings and early ambulation were encouraged to prevent DVT/ PE. Most studies evaluating the role of tranexamic acid in THA have used chemoprophylaxis against DVT/ PE and have reported that the use of tranexamic acid was not associated with a significant increase in the risk of DVT/PE [4, 10, 26]. Since the chemoprophylaxis may counter the hypercoagulability effect of tranexamic acid, it is a confounding factor. However, by not using chemoprophylaxis against DVT/PE, our study conclusively shows that the use of tranexamic acid is not associated with an increased risk of DVT/PE. The preoperative and postoperative day 5 screening for DVT using venous Doppler ensured that asymptomatic DVTs were not missed. The limitation of our study was the small sample size that may affect the results of the present trial. A multicenter, randomized study with large sample size may yield more statistically significant results.

## Conclusion

The present analysis needs to be put in the perspective of the rapidly increasing number of hip arthroplasties being performed each year. Our findings suggest that a single pre-incisional dose of tranexamic acid results in a statistically significant and clinically meaningful reduction in intraoperative, postoperative, and total blood loss from uncemented THA for osteonecrosis of femoral head without increasing the risk of prothrombotic complications.

## Data Availability

The data that support the findings of this study are available from [Shalby Hospitals India] but restrictions apply to the availability of these data, which were used under license for the current study, and so are not publicly available. Data are however available from the authors upon reasonable request and with permission of [Shalby Hospitals India].

## Abbreviations

BMI: Body Mass Index
DVT: Deep Vein Thrombosis
Hb: Haemoglobin
NSAIDs: Nonsteroidal Anti-inflammatory Drugs
PE: Pulmonary Embolism
THA: Total Hip Arthroplasty
TXA: Tranexamic Acid
VTE: Venous Thrombo-Embolism
